# Highly Sensitive Sphere-Tube Coupled Photoacoustic Cell Suitable for Detection of a Variety of Trace Gases: NO_2_ as an Example

**DOI:** 10.3390/s22010281

**Published:** 2021-12-30

**Authors:** Zhengang Li, Ganshang Si, Zhiqiang Ning, Jiaxiang Liu, Yonghua Fang, Beibei Si, Zhen Cheng, Changping Yang

**Affiliations:** 1 Key Laboratory of Environmental Optics and Technology, Anhui Institute of Optics and Fine Mechanics, Hefei Institutes of Physical Science, Chinese Academy of Sciences, Hefei 230031, China; lzgyx@mail.ustc.edu.cn (Z.L.); gssi@aiofm.ac.cn (G.S.); ningzq@mail.ustc.edu.cn (Z.N.); luckuis@aiofm.ac.cn (J.L.); sibeibei@mail.ustc.edu.cn (B.S.); cz199788@mail.ustc.edu.cn (Z.C.); cpyang@mail.ustc.edu.cn (C.Y.); 2 University of Science and Technology of China, Hefei 230026, China

**Keywords:** photoacoustic spectroscopy, photoacoustic cell, long optical path, acoustic resonance, resonance mode, finite element simulation

## Abstract

The concentration of trace gases in the atmospheric environment is extremely low, but it has a great impact on the living environment of organisms. Photoacoustic spectroscopy has attracted extensive attention in the field of trace gas detection because of its high sensitivity, good selectivity, and fast response. As the core of a photoacoustic detection setup, the photoacoustic cell has a significant impact on detection performance. To improve detection sensitivity, a sphere-tube coupled photoacoustic cell (STPAC) was developed, which was mainly composed of a diffuse-reflective sphere and an acoustic resonance tube. Modulated light was reflected multiple times in the sphere to increase optical path, and photoacoustic (PA) signals were further amplified by the tube. Based on STPAC, a PA gas detection setup was built with a laser diode (LD) at 450 nm as the light source. The experimental results showed that the minimum detection limit (noise equivalent concentration, NEC) of NO_2_ was ~0.7 parts per billion (ppb). Compared with the T-type PA cell (TPAC) in which the modulated light passed through the sphere, the signal-to-noise ratio of STPAC was increased by an order of magnitude at the same concentration of the NO_2_ sample.

## 1. Introduction

Nitrogen dioxide (NO_2_) is a trace gas toxic to living beings, which is mainly discharged into the atmosphere by combustion and other processes [1,2]. The average concentration of NO_2_ in the atmosphere is usually 5–30 ppb, but the concentration is several orders of magnitude higher near the NO_2_ release source [3]. Due to the influence of sunlight, NO_2_ decomposes into NO and oxygen free radicals, resulting in an increase of O_3_ in the air [4]. NO_2_ has a strong absorption line in the visible region, and absorption intensity is the largest in the blue-violet range [5,6]. However, when the wavelength is lower than 415 nm, NO_2_ undergoes photolysis [7,8]. With the development of laser diode (LD) manufacturing technology, a low-cost blue LD with a central wavelength of 450 nm has become a suitable light source for the detection of NO_2_ by absorption spectroscopy.

As an indirect absorption spectroscopy technology, photoacoustic spectroscopy (PAS) has the advantages of high sensitivity, good selectivity, fast response, and enclosure in a compact module [9,10,11,12,13]. It is widely used in power detection, medical diagnosis, industrial control, atmospheric monitoring, and combustion analysis [14,15,16,17,18]. In recent years, many researchers have used photoacoustic technology to achieve the detection of NO_2_. Bernhardt introduced an LED-excited photoacoustic device for NO_2_ measurement. Out-of-phase signals produced in two resonators achieved a larger signal and common mode noise rejection, which made it possible to measure NO_2_ down to 60 ppb [6]. Zheng designed a method to suppress the background noise caused by stray light of QEPAS NO_2_ sensor, achieving ppb level detection of NO_2_ [19]. Yin developed a sub-ppb level photoacoustic sensor by using a 3.5 W laser diode and a differential photoacoustic cell; the PA cell was used to match the imperfect laser beam and reduce the external noise [20].

Detection sensitivity of PAS is affected by the cell constant of the PA cell, incident light power, microphone sensitivity and other factors [21]. As the core unit of the PA detection device, an effective design of the PA cell can improve the sensitivity [22]. PA cells are divided into nonresonant and resonant types according to whether they can amplify the PA signal [23,24]. At present, common resonant PA cells include Helmholtz, H-type, T-type, and their variants [25,26,27,28,29]. Helmholtz PA cells have lower resonance frequency and weaker ability to amplify the acoustic signal than H-type and T-type PA cells. Compared with H-type PA cells (HPAC), the volume of T-type PA cells (TPAC) is reduced by nearly half at the same resonance frequency. For the traditional TPAC, modulated light passes through the absorption cell, and the optical path is only the length of the cell.

It is known that the intensity of PA signal is affected by incident light power. With the improvement of light power, the PA signal also increases, but high-power light sources usually have high cost and large volumes. Some researchers have used devices in which light beams are reflected multiple times to achieve the equivalent effects [30,31,32,33,34]. Lassen reported a photoacoustic sensor based on an integrating sphere. The light beams were reflected multiple times in the sphere, and a fixed length tube was used to amplify the PA signal. The detection of NO_2_ was realized by using a blue LED with a central wavelength of 415 nm, and the minimum detectable concentration was 1.9 ppm [35]. Chen proposed a PA sensor which worked in a nonresonant state, and light beams were reflected multiple times on the inner wall of the PAC. When average time was 400 s, the limit of detection of C_2_H_2_ was ~31 parts per billion (ppb) [36]. Yang developed an enhanced fiber PA sensor. For improving the PA signal, an incident laser was reflected on the inner surface of a ring, and the minimum detection limit of C_2_H_2_ was ~23.6 ppb [37]. Jin introduced a PA sensor based on a mirror with high reflectance. Compared with a traditional PA system, the response capacity of NO_2_ was increased from 0.016 µV/ppb to 0.2562 µV/ppb [38]. Qiao developed a multi-pass quartz-enhanced PA sensor. The light beams passed through prong spacing of the quartz six times, which improved the PA signal ~3.2 times [39]. According to the above research, improving the number of light beam reflections can increase the optical path and effectively amplify the PA signal. The combination of multiple reflection and acoustic resonance proposed by Lassen [35] was an effective way to improve the photoacoustic signal. However, the effects of different tube lengths and resonance modes on photoacoustic signals were not analyzed. In fact, tube length affects the resonance frequency and the ability of amplifying the PA signal. Therefore, the optimization of tube length is of great significance to improve the signal-to-noise ratio (*SNR*).

Based on the above analysis, a sphere-tube coupled photoacoustic cell (STPAC) was designed. Instead of the cylindrical absorption cell for STPAC, a spherical absorption cell was employed made of high reflectance polytetrafluoroethylene (PTFE). Modulated light was reflected multiple times on the inner wall of the sphere, which was similar to an integrating sphere. The sphere was wrapped in two aluminum hemispheres to improve heat dissipation capacity and reduce the negative impact of the solid-state photoacoustic effect caused by absorbing light energy. To amplify the PA signals, an acoustic resonance tube was connected to the sphere, and the inner wall of the tube was blackened to reduce the influence of stray light. Sound pressure of acoustic resonance tubes with different lengths and resonance modes were simulated by finite element analysis. According to the simulation results, three special lengths of acoustic resonance tubes were processed, and the optimal tube length was obtained by experiments. To prevent the photolysis of NO_2_, a 450 nm LD was used as the excitation light source. The experimental results showed that the minimum detection limit of NO_2_ reached the sub-ppb level.

## 2. Theory and Simulation

### 2.1. Theory of Photoacoustic

PAS gas detection is an indirect absorption spectroscopy technology that calculates gas concentration by detecting the acoustic signal generated by the gas absorbing light energy. When gas molecules absorb periodically modulated light, energy level transitions occur resulting in gas molecules changing from the ground state to the excited state. Excited molecules release heat energy by collision, which causes a periodic change of pressure in the closed PA cell. The pressure produces sound waves with the same frequency as the modulated light, which are called PA signals [40].

If the intensity of the modulated light is I(r,t) and the absorption coefficient of the gas to be measured is $\alpha_{p}$, the heat density source H(r,t) formed after the gas molecules absorbing the light energy can be expressed as Equation (1) [41]:

(1) $$H\left(r,t\right)=\alpha_{p}I\left(r,t\right).$$

The heat density source vibrates the gas in the PA cell and excites an acoustic signal. It is assumed that the inner surface of the PA cell is rigid and there is no velocity component perpendicular to the wall. When ignoring the loss of gas molecules, the nonuniform wave equation of sound pressure in the cylindrical PA cell is [41,42,43]:

(2) $$\frac{\partial^{2}p\left(r,t\right)}{\partial t^{2}}-c^{2}\nabla^{2}p\left(r,t\right)=\left(\gamma -1\right)\frac{\partial H\left(r,t\right)}{\partial t}$$

In Equation (2), p represents the sound pressure, c is the sound velocity of the gas in the PA cell, γ is the adiabatic coefficient of the gas. Equation (3) [44,45] can be obtained by Fourier transform of Equation (2):

(3) $$\left(\nabla^{2}+\frac{\omega^{2}}{c^{2}}\right)p\left(r,\omega \right)=\frac{\gamma -1}{c^{2}}i\omega H\left(r,\omega \right)$$

where ω is the modulated angular frequency. The expression of p(r,ω) is [46]:

(4) $$p\left(r,\omega \right)=\sum A_{j}\left(\omega \right)p_{j}\left(r\right),$$

where $p_{j}\left(r\right)$ is the solution of the normal mode; $A_{j}\left(\omega \right)$ is the mode amplitude. For the PA cell with a regular shape, such as a cylindrical type, the mode amplitude expression is [47]:

(5) $$A_{j}(\omega )=-\frac{i\omega }{\omega_{j}^{2}}\frac{\alpha \left(\gamma -1\right)\int p_{j}^{*}IdV}{V_{c}\left[1-\frac{\omega^{2}}{\omega_{j}^{2}}-i\left(\frac{\omega }{\omega_{j}Q_{j}}\right)\right]}$$

where $\omega_{j}$ is the resonant angular frequency in the normal mode; $Q_{j}$ is the quality factor and $V_{c}$ is the volume of the PA cell. A special case is considered. If the modulated light I does not change with spatial location r (I(r,ω)=I(ω)), when j≠0, $\int p_{j}^{*}IdV=0$. The only nonzero mode is $p_{0}$, and the resonant angular frequency $\omega_{0}$ is 0. Therefore, the sound pressure in the PA cell is independent of r. The mode amplitude can be expressed as [24]:

(6) $$A_{0}\left(\omega \right)=\frac{i\alpha \left(\gamma -1\right)I}{\omega \left(1-\frac{i}{\omega \tau_{0}}\right)}$$

where $\tau_{0}$ is the damping time of $p_{0}$. It can be seen from Equation (6) that with an increase of modulated angular frequency ω, the mode amplitude decreases, and the PA cell works in the nonresonant state.

According to Equation (5), the mode amplitude $A_{j}\left(\omega \right)$ reaches a maximum when the modulated angular frequency ω is equal to the resonant angular frequency $\omega_{j}$, and the PA cell works in resonant state. If the wavelength of the sound wave is greater than the cross-section size of the resonator, such as in a slender tube, only a one-dimensional longitudinal sound field along the length direction is generated. When both ends of the tube are open, such as in the HPAC, the equation of the first-order longitudinal resonance frequency can be expressed by Equation (7). When one end of the tube is closed and the other end is open, such as in the TPAC, Equation (8) applies [48,49]:

(7) $$f_{H}=\frac{c}{2\left(L+\frac{16}{3\pi }R\right)}$$

(8) $$f_{T}=\frac{c}{4\left(L+\frac{8}{3\pi }R\right)}$$

where L and R represent the length and radius of the tube, respectively. The relevant characteristics of the PA cell, such as structure, material, and size, are regarded as constant $C_{\mathrm{cell}}$. When the optical power of the incident light is $P_{0}$; the microphone sensitivity is $M_{s}$ and the gas concentration is $C_{g}$. The PA signal can be expressed as [50]:

(9) $$S_{{}^{{}_{\mathrm{PA}}}}=\alpha_{p}P_{0}C_{\mathrm{cell}}M_{s}C_{g}$$

when the PA cell works in the resonant mode, Equation (9) can be rewritten as [25]:

(10) $$S_{\mathrm{PA}}=\frac{\left(\gamma -1\right)LQ}{V_{c}\omega }M_{s}P_{0}\alpha_{p}$$

where L is the gas absorption path. According to the principle of absorption spectroscopy, when the incident light power $P_{0}$ is constant, the PA signal is directly proportional to the gas absorption path. The gas absorption path (equivalent to the optical path) can be increased by using the integrating sphere as the absorption cell. The equation of the equivalent optical path in the sphere is [32]:

(11) $$L_{eq}=\frac{2D}{3(1-\rho )}$$

where ρ is the average reflectance and D is the diameter of the sphere.

### 2.2. Simulation and Design

**Figure 1:**
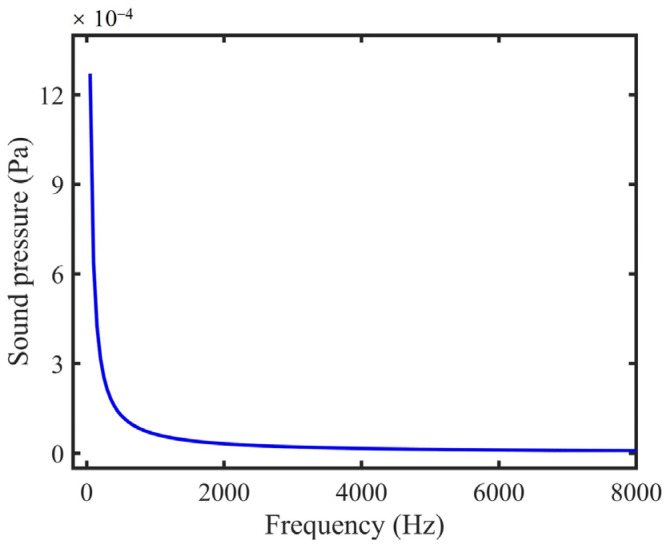
Simulated sound pressure curve at any position on the inner surface of the integrating sphere in the nonresonant state.

Due to the characteristics of the integrating sphere, the light field in the sphere was uniform. The uniform light field produced homogeneous heat in the device. The sound pressure at any point on the inner surface of the sphere was simulated. A sphere with a diameter of 5.08 cm was set as a uniform heat source with a value of 1 W/m^3^. As shown in Figure 1, the sound pressure was inversely proportional to the modulated frequency, which is consistent with Equation (6). Therefore, there was no resonance in the sphere.

**Figure 2:**
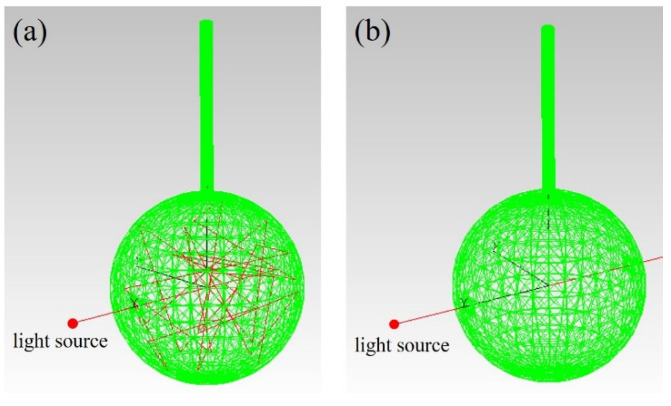
Simulated light field distribution of (**a**) STPAC and (**b**) TPAC.

For the sake of combining long optical path and acoustic resonance, a tube was connected to the integrating sphere. As an example, the length and diameter of the tube were 5 cm and 4 mm, respectively. To reduce the noise caused by the reflection of light beams in the tube, the inner wall of the tube was blackened. The light field distribution was also simulated. A collimated light beam was reflected multiple times in STPAC, as shown in Figure 2a. When the light beam passed through the sphere, it was similar to TPAC, as shown in Figure 2b. In fact, the light inlet, light outlet, air inlet, and outlet could not reflect the light beam. However, when the opening of the sphere was less than 5% of the inner surface area of the integrating sphere, the influence of the diffuse reflection effect could be ignored [51], and was not considered in the simulation. Only the light beam in which the laser energy is greater than 90% of the initial energy is shown in Figure 2a.

**Figure 3:**
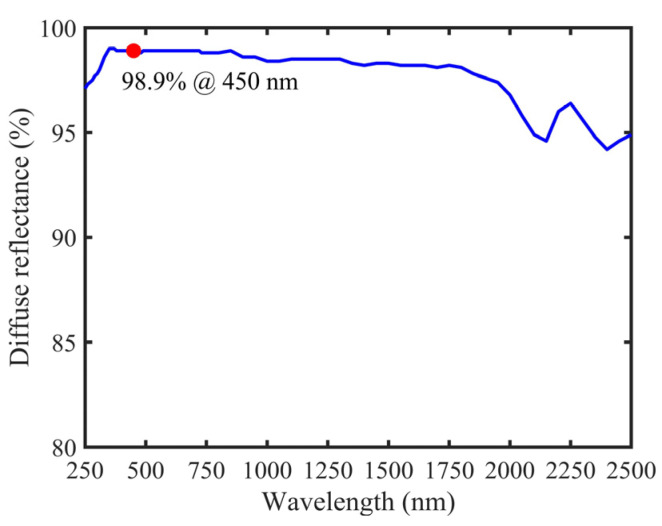
Diffuse reflectance curve of PTFE.

The diffuse reflectance curve of PTFE (National Institute of Metrology, CHINA. Certificate No. GXcl2021-00129) is shown in Figure 3; the corresponding value at 450 nm was 98.9%. The equivalent optical path of STPAC calculated by Equation (11) was ~308 cm, which was 60 times that of TPAC (~5.08 cm).

**Figure 4:**
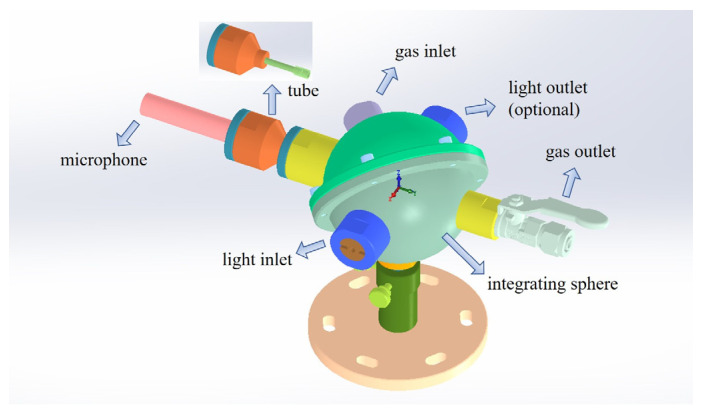
3-D model of STPAC.

The 3-D model of STPAC is shown in Figure 4, and is mainly composed of light inlet, light outlet (optional), gas inlet, gas outlet, acoustic resonance tube and integrating sphere. The light outlet could be configured into ‘open’ or ‘closed’ modes, so that the PA cell could be flexibly converted between STPAC and TPAC. Due to the threaded structure of the joint, the PAC was conveniently coupled with different length tubes.

One end of the tube was coupled with the sphere and a microphone was installed at the other end, corresponding to the state of ‘open’ and ‘closed,’ respectively. In most cases, the first-order longitudinal resonance frequency could be approximately calculated by Equation (8). However, to prevent the diaphragm at the end of the microphone from being worn during installation, a cylindrical gap with a length of 2 mm and a diameter of 12.3 mm was reserved between the microphone and the end of the tube. Therefore, for the STPAC developed in this paper, there would be an error between the resonance frequency calculated by Equation (8) and the actual resonance frequency. With the development of the numerical calculation, the resonance frequency of an irregular PA cell could be obtained by finite element simulation.

**Figure 5:**
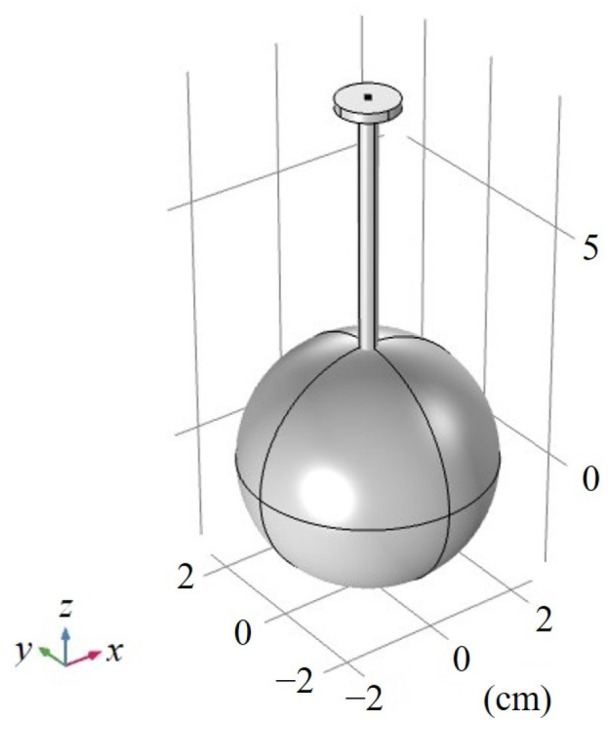
Finite element simulation model of STPAC.

For accelerating simulation speed and reducing computational complexity, the simulation model of STPAC was simplified. The less influential parts were removed, including gas inlet, gas outlet, light inlet, and light outlet, as shown in Figure 5.

**Figure 6:**
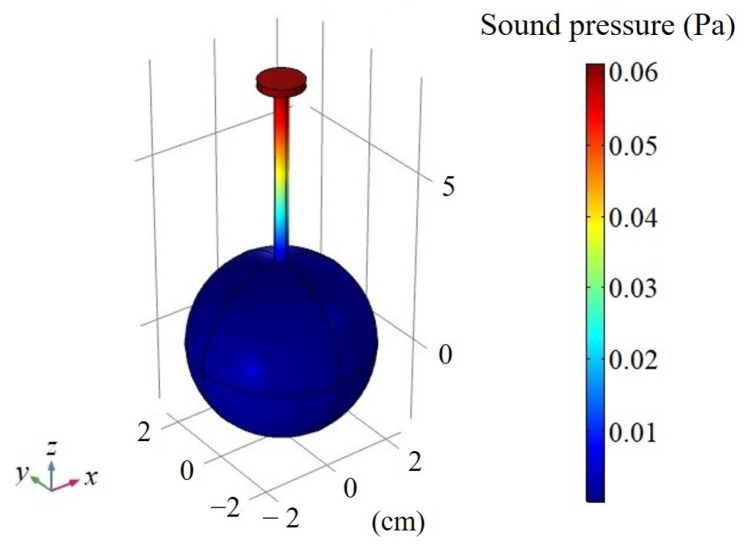
Simulated sound pressure distribution in the first-order longitudinal resonance mode of STPAC.

The resonance frequency is the natural frequency of the photoacoustic cell, which is independent of the excitation mode of the light beam. Therefore, a sphere of 5.08 cm in diameter was set as a uniform heat source with a value of 1 W/m^3^. When the tube length was 5 cm, the sound pressure distribution of STPAC in the first-order longitudinal resonance mode was as shown in Figure 6. The resonance frequency was 1238 Hz, and the maximum sound pressure was located at the end of the tube connected to the microphone. At the same time, the sound pressure in the integrating sphere was close to zero.

**Figure 7:**
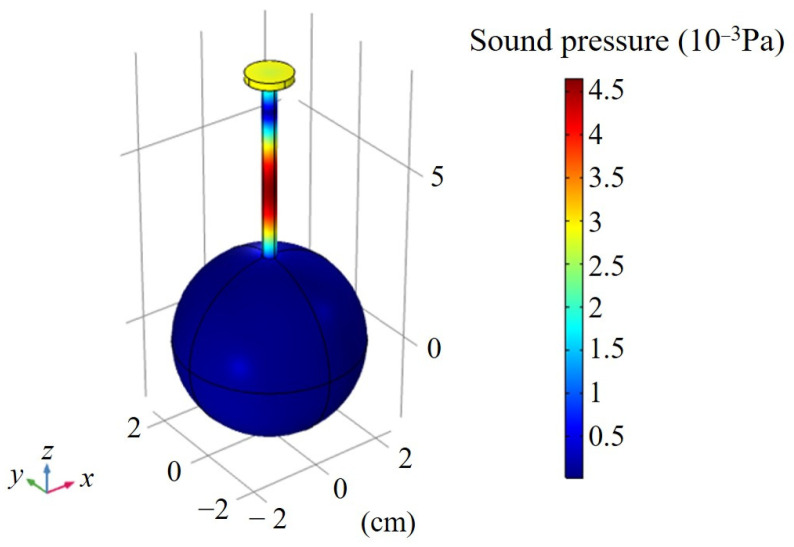
Simulated sound pressure distribution in the second-order longitudinal resonance mode of STPAC.

**Figure 8:**
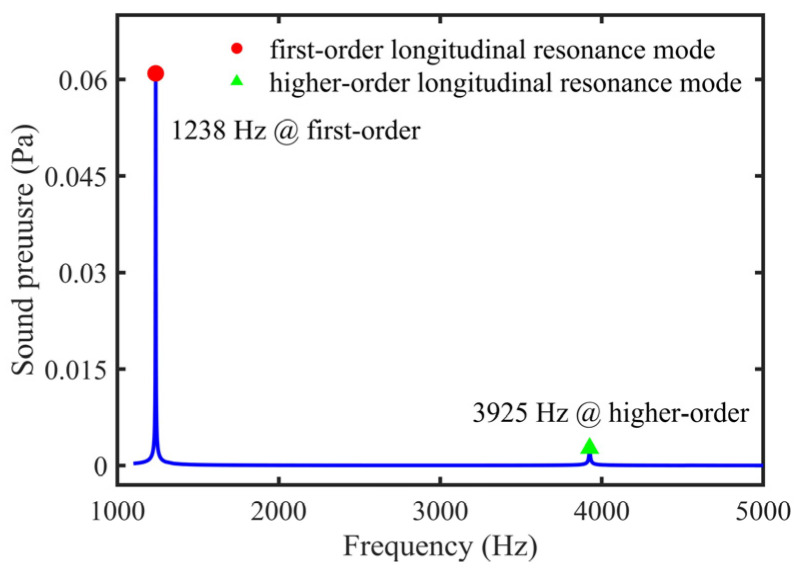
Simulated variation curve of the sound pressure at the end of the tube with frequency.

With an increase of frequency, the second-order longitudinal resonance mode had a resonance frequency of 3925 Hz in the tube, as shown in Figure 7. The variation curve of sound pressure at the end of the tube with frequency is shown in Figure 8. When the resonance order was raised, the sound pressure at the end of the tube decreased. Therefore, the tubes described in this paper worked in the first-order longitudinal resonance mode to produce maximum sound pressure.

**Figure 9:**
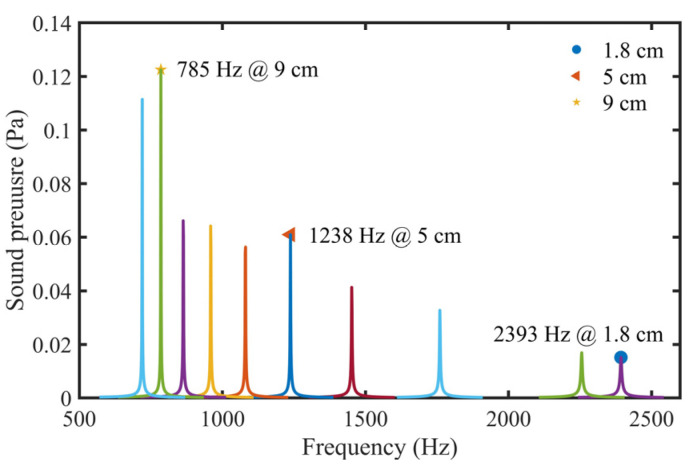
Simulated sound pressure curves of STPAC with different tube lengths. Tube lengths from left to right were 10 cm, 9 cm, 8 cm, 7 cm, 6 cm, 5 cm, 4 cm, 3 cm, 2 cm, and 1.8 cm, respectively.

To analyze the influence of different tube lengths on the photoacoustic signal, sound pressure curves of STPAC with different tube lengths were simulated, as shown in Figure 9. The sound pressure of the 9 cm tube was the largest. Sound pressures of 5 cm, 6 cm, 7 cm, and 8 cm tubes were 0.0610 Pa, 0.0563 Pa, 0.0642 Pa and 0.0661 Pa, respectively. So, the sound pressure of the 5 cm tube was 108, 95 and 92% of the other three tubes. However, the resonance frequency was higher, and the volume was smaller. Meanwhile, 1.8 cm was the shortest tube that could be installed in the photoacoustic cell. Therefore, 1.8 cm, 5 cm and 9 cm acoustic resonance tubes were processed for subsequent experiments.

## 3. Experiments and Results

**Figure 10:**
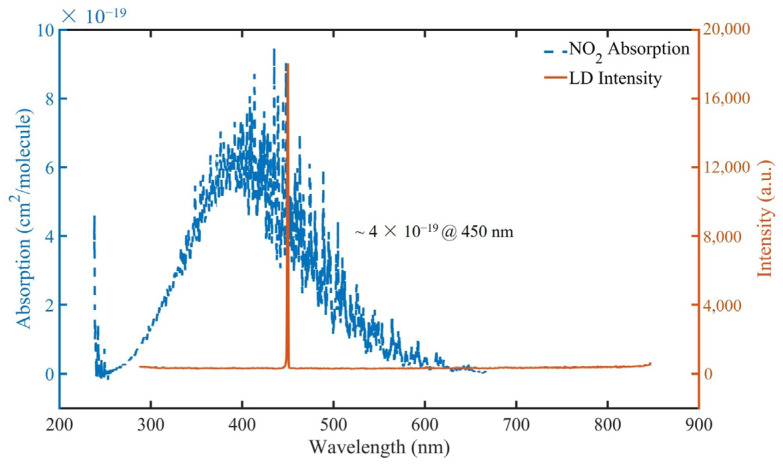
Emission spectrum of LD and absorption spectrum of NO_2_ (Ref. [52], copyright obtained from Elsevier).

A PA gas detection setup was built to verify the performance of STPAC. To avoid photolysis of NO_2_, a laser diode (JLM45160ZMW, Dongguan Blueuniverse Laser, Dongguan, China) with a central wavelength of 450 nm, line width of 4 nm and a light intensity of 500 mW was selected as the excitation light source. Since the average transmittance at 450 nm was ~93.5%, the average incident light power was ~468 mW. The emission spectrum of LD was measured by a spectrometer, as shown in Figure 10.

**Figure 11:**
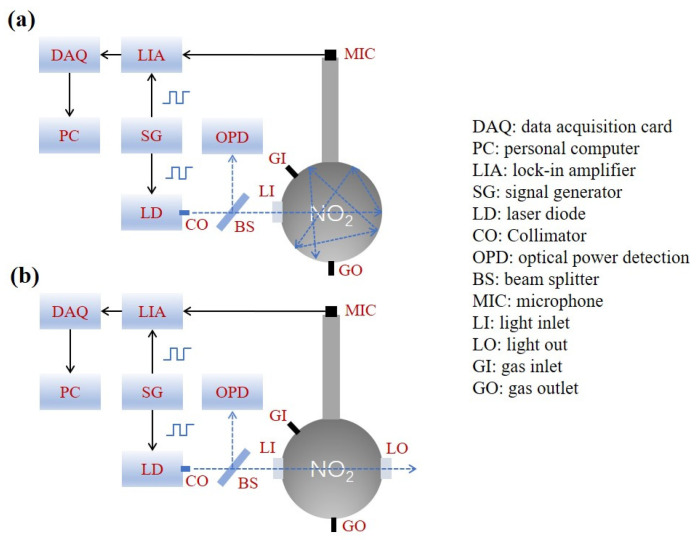
Schematic diagram of PA gas detection setup; (**a**) STPAC, (**b**) TPAC.

The laser entered STPAC through the optical window. The NO_2_ samples were commercial standard gases composed of different concentrations of NO_2_ and N_2_. To replace the gas in the STPAC, a flow rate of 1 L/min was employed. After the gas replacement was complete, the gas inlet valve and gas outlet valve were closed. To reduce the noise caused by gas movement, the PA experiments were carried out after 10 s. A signal generator was used to provide a signal for intensity modulation of LD. To detect PA signals, a microphone (MPA201, BSWA) with a sensitivity of 50 mV/Pa was installed at the end of the tube. The PA signals were demodulated by a lock-in amplifier, and the integration time was set to 1 s. The demodulated signals were collected by the data acquisition card and uploaded to a computer for analysis. The schematic diagrams of the setup are shown in Figure 11.

**Figure 12:**
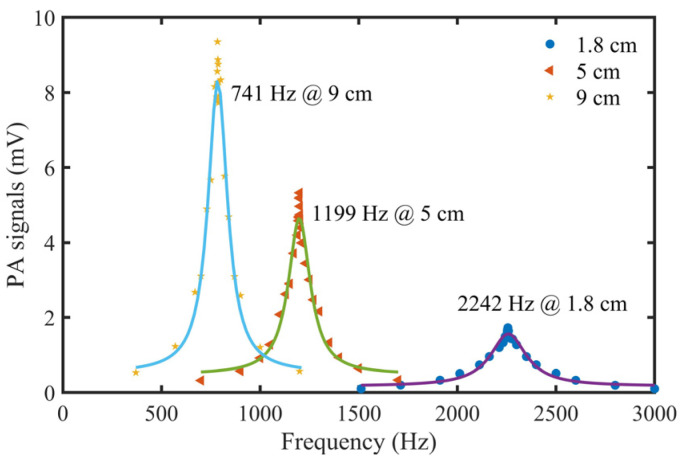
Measured PA signals curves of STPAC with different tube lengths.

Due to the difference between the simulation and the actual situation, it was necessary to calibrate the actual first-order longitudinal resonance frequency of STPAC through experiments. The gas to be measured was commercial standard 10 parts per million (ppm) NO_2_/N_2_. Sound pressure (PA signals) data of the tubes with different lengths were recorded, and the data were fitted using the Lorentz equation. The results are shown in Table 1 and Figure 12. Compared with Figure 9, the simulated sound pressure and measured PA signals had similar trends. The inner wall of the tube was not precisely polished, the thermal viscosity loss and boundary loss were relatively large, so the Q values were slightly low.

For comparing the PA signals in the first-order and high-order longitudinal resonance modes, a 5 cm tube was taken as an example. The resonance frequency in the high-order was 3560 Hz, and the PA signal was 0.223 mV. The PA signal in the first-order was 5.345 mV, which was ~24 times higher than the second-order PA signal. The simulation results show that the simulated sound pressure of the first-order and second-order were 0.061 Pa and 0.0027 Pa; the former was ~22.4 times higher than the latter. The simulated and measured results were approximately consistent, as shown in Table 2. Because of the higher sound pressure, the first-order longitudinal resonance mode was used in this paper.

**Figure 13:**
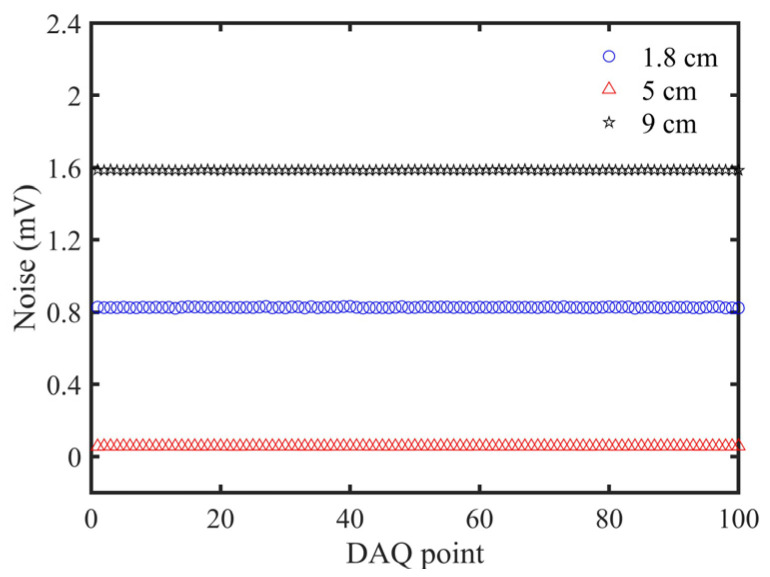
Noise distribution of STPAC with different length tubes: 1.8 cm, 5 cm, and 9 cm.

Noise distribution of STPAC with different length tubes in the first-order longitudinal resonance mode was analyzed with the pure N_2_ background. The LD was turned on, and the resonance frequency corresponding to different tube lengths was used to modulate the LD. Experimental results are shown in Figure 13.

To analyze the detection performance of tubes with different lengths, the signal-to-noise ratio (*SNR*) was used as the evaluation standard, and its calculation formula was [20]:

(12) $$SNR=\frac{Signal-\mu (B)}{\sigma },$$

where Signal is the measured PA signal, σ is the noise deviation, and μ(B) is the average value of noise. The calculation equation of σ is:

(13) $$\sigma =\sqrt{\frac{1}{n}\sum_{k=1}^{n}{\left(B_{k}-\mu \left(B\right)\right)}^{2}},$$

where $B_{k}$ is measured value of noise, and n is the total number of noise samples.

The *SNR* of different length tubes is shown in Table 3. The PA signal generated by the 9 cm tube was the largest, but the resonance frequency was relatively low. Because of the influence of 1/f noise, electronic noise, and ambient noise, the total noise value would increase if frequency was reduced [22,23]. Since the inner walls of the three tubes were blackened, stray light was absorbed by the inner wall of the tubes and was not reflected again. Therefore, the solid-state photoacoustic effects produced by the tubes were almost equal theoretically. However, the length of the 1.8 cm tube was too short, and some stray light might not be absorbed by the inner wall of the tube but could directly irradiate the diaphragm of the microphone, resulting in thermal noise and large noise fluctuation. Due to the moderate length and high resonance frequency, the *SNR* of the 5 cm tube was the highest, and was ~2.7 times of 9 cm tube; so this was used in subsequent experiments.

**Figure 14:**
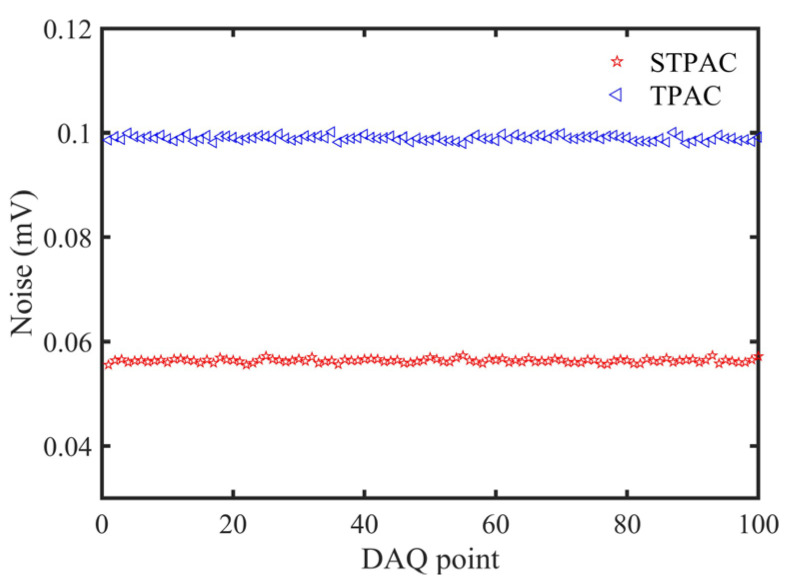
Noise distribution of STPAC and TPAC.

STPAC was compared with TPAC to verify the ability of amplifying PA signals. The noise distribution is shown in Figure 14. The average noise value and standard deviation of STPAC were both lower than TPAC, and the performance parameters are shown in Table 4. The *SNR* of STPAC was ~16 times that of TPAC.

Because the modulated light passed through two optical windows of TPAC (transmittance of calcium fluoride window at 450 nm was ~93.5%), ~12.6% of the light energy was absorbed to produce noise with the same frequency as the PA signals. At this time, if other noises were ignored, the main noise came from the heat energy absorbed by the two windows. From Table 4, if the noise caused by the first window was *X*, the equation *X* + 0.935 *X* = 0.099 was obtained and *X* = 0.0512 could be calculated, and was close to the noise of STPAC (0.0563 mV). Therefore, the noise absorbed by PTFE material was 0.0563–0.0512 = 0.0051 mV, which was only 10% of the window noise. Although the light energy of STPAC was all absorbed inside the integrating sphere, which was wrapped by two aluminum hemispheres of high heat dissipation, the absorbed light energy quickly diverged to the outside in the form of heat. So, the noise of STPAC was much lower. At the same time, the microphone was located at the end of the 5 cm tube away from the spherical absorption cell, so that the noise caused by stray light was at a low level. It was expected that the noise of STPAC could be reduced to a lower level after optimizing the thickness of the hemispherical shells, selecting materials with the higher heat transfer coefficient, and replacing a window with a higher transmittance.

**Figure 15:**
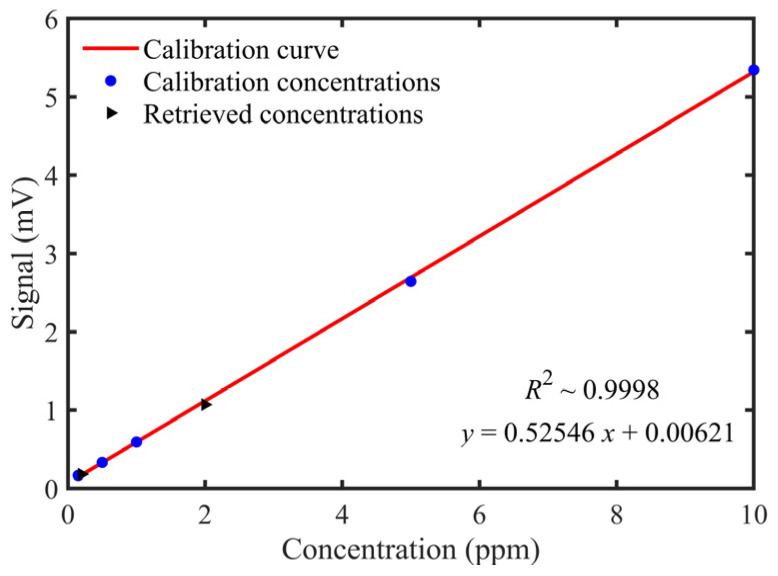
NO_2_ concentration calibration curve of STPAC.

A series of commercial standard NO_2_/N_2_ samples with concentrations of 0.15, 0.5, 1, 5 and 10 ppm were used to calibrate the PA gas detection setup based on STPAC. The fitted concentration-signal curve was y=0.52546x+0.06621, and *R*^2^ was ~0.9998. The accuracy of the setup was verified by using 0.25 ppm and 2 ppm commercial standard NO_2_/N_2_ samples. The related errors between the retrieved concentrations (0.26 ppm and 0.91 ppm) by setup and the actual concentrations (0.25 ppm and 2 ppm) were 4% and −4.5% respectively, as shown in Table 5 and Figure 15. The experimental results showed that the response capacity of the setup was 0.52546 mV/ppm for 0–10 ppm NO_2_. The minimum detection limit (noise equivalent concentration, NEC) of NO_2_ was ~0.7 ppb calculated by using 1 time σ.

**Figure 16:**
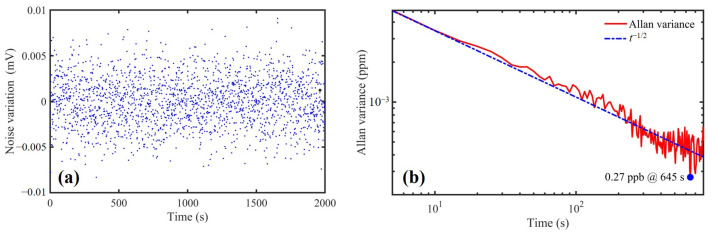
(**a**) Noise variation and (**b**) Allan variance curve of the setup with long-time measurement.

According to the Allan variance study, when the average time was long enough, high sensitivity could be achieved by the setup. To analyze the minimum detection sensitivity of the setup, pure N_2_ was flushed into the STPAC for long-time detection, and the Allan variance of noise equivalent concentration was used to evaluate stability, as shown in Figure 16. When the average time was 645 s, the minimum detection sensitivity was ~0.27 ppb.

## 4. Discussion

**Figure 17:**
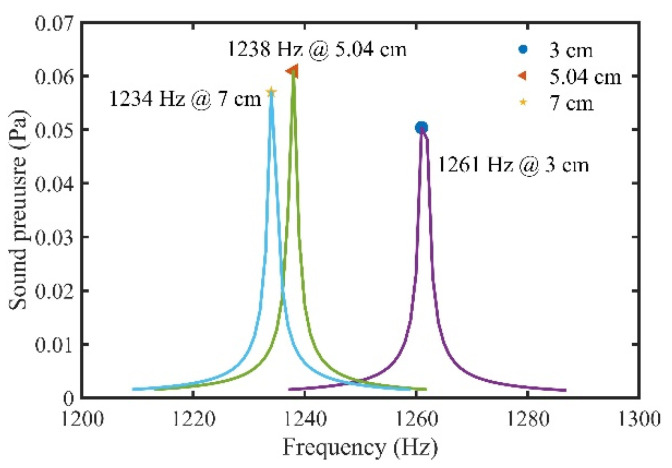
Simulated sound pressure curves of STPAC with different sphere sizes.

In this paper, a commercial diffuse sphere with a diameter of standard size 5.08 cm (2 in) and an acoustic resonance tube of 5 cm were used, which preliminarily verified the feasibility of STPAC to increase the sensitivity of photoacoustic detection. We focused on the influence of tube length and resonance mode on photoacoustic detection ability. However, the size of the sphere also affects the optical path and sound pressure. The diameters of 3 cm, 5.04 cm and 7 cm spheres, and a 5 cm tube were taken as examples. The volume, resonance frequency, sound pressure and equivalent optical path (at 450 nm) of the three spheres were analyzed, as shown in Table 6 and Figure 17. Compared with a 7 cm sphere, the 5.08 cm sphere had a 28% reduction in optical path but a 7% increase in sound pressure and a 62% reduction in volume. Therefore, considering the optical path and sound pressure, STPAC could obtain a longer optical path and higher sensitivity by carefully selecting the size of the sphere.

To verify the improvement of the detection performance of STPAC for other gases, CO_2_ was taken as an example, and the wavelength was chosen as 2004 nm. The excitation light source was a 3 mW distributed feedback laser with intensity modulation, and the gas to be measured was commercial standard 1000 ppm CO_2_/N_2_. Figure 3 shows that the reflectance of PTFE near 2000 nm was ~96.8%, and the equivalent optical path was ~106 cm according to Equation (11). Compared with TPAC (5.04 cm), the optical path of STPAC was increased by ~20 times. The experimental results are shown in Table 7. Compared with TPAC, the signal-to-noise ratio of STPAC for CO_2_ gas samples was increased by ~5 times (the minimum detection limit was reduced by ~5 times). Therefore, in the high reflectance band of PTFE (250–2500 nm, reflectance > 94.2%), the detection ability of STPAC for other gas samples could be also improved.

**Figure 18:**
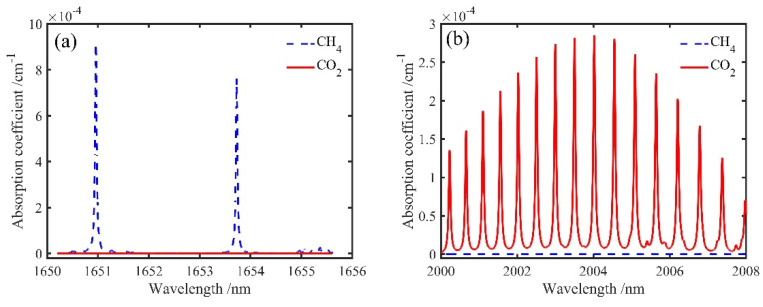
Simulated absorption lines of CH_4_ and CO_2_ near (**a**) 1653 nm and (**b**) 2004 nm.

Due to the good selectivity of photoacoustic technology, STPAC could also be used for the detection of mixed gas. First, from the point of view of the light source, mutual interference between mixed gases could be avoided if an appropriate excitation spectral line was selected [53]. A CH_4_ and CO_2_ mixture was taken as an example, and the absorption lines of 2000 ppm CH_4_ and 2000 ppm CO_2_ near 1653 nm and 2004 nm were simulated through HITRAN database, as shown in Figure 18. At 1653 nm (CH_4_ absorption peak) and 2004 nm (CO_2_ absorption peak), CH_4_ and CO_2_ did not interfere. Second, detection of the mixed gas could be realized by using frequency division multiplexing [54] or time division multiplexing technology [55]. Therefore, as a long optical path photoacoustic absorption cell, STPAC would be suitable for the detection of mixed gas.

## 5. Conclusions

In this study, a STPAC for PA gas detection was developed. An integrating sphere was used as the absorption cell, and the modulated light reflected multiple times to increase the optical path. Compared with the TPAC, in which modulated light passed through the absorption cell, the optical path was increased by ~60 times. Because the light field in the integrating sphere was uniform and did not produce resonance, an acoustic resonance tube was coupled with the sphere to produce a specific resonance mode. The sound pressure of different lengths of tubes in first-order longitudinal resonance mode was simulated, and three special tube lengths were processed. According to the simulation and experimental results, although the 9 cm tube produced the largest signal, its resonance frequency was low and the noise was relatively large, so the *SNR* was not optimal. The length of the 5 cm tube was moderate, and the higher resonance frequency suppressed the noise, so the *SNR* was the highest, and ~2.7 times that of 9 cm tube.

The performance of the PA gas detection setup based on STPAC was analyzed by using NO_2_ gas samples. A low-cost LD with wavelength of 450 nm was selected as the excitation light source, the PA signal was excited by intensity modulation, and the signal was collected by a microphone located at the end of the tube away from the spherical absorption cell. In the range of 0–10 ppm, the PA signals had a fine linear relationship with NO_2_ concentrations, *R*^2^ was ~0.9998, and the response capacity was 0.52546 mV/ppm. The relative errors between the retrieved concentrations and the actual concentrations were within ±5%. Because of the two aluminum hemispherical shells with high heat transfer, the light energy absorbed by the integrating sphere was converted into heat energy and quickly diverged to achieve a low level of noise. At the same time, the blackened inner wall of the tube reduced the noise caused by stray light. When *SNR* was 1, the minimum detection limit (NEC) of the setup was calculated to be ~0.7 ppb, which was an order of magnitude lower than TPAC. At the same time, the PA signal intensity of STPAC was also an order of magnitude higher than that of TPAC. Allan variance was used to evaluate the stability of the setup with long-time measurement. When the average time was 645 s, the minimum detection sensitivity reached ~0.27 ppb. 

In conclusion, compared with the traditional TPAC, STPAC combined long optical path and acoustic resonance without adding additional volume to achieve a lower detection limit and increased the *SNR* by ~16 times. It was expected that STPAC would be able to detect various trace gases with absorption peaks in the high reflectance band (250–2500 nm) of the diffuse reflective material PTFE. Because the photoacoustic signal was affected by the volume of the photoacoustic cell, the size of the absorption cell will be further optimized in future work to achieve higher sensitivity detection with a smaller volume PA cell.

## Figures and Tables

**Table 1:** Frequency calibration data of tubes with different lengths of STPAC.

Tube Length (cm)	Center Frequency (Hz)	Full Width at Half Maximum (Hz)	*R*^2^ (%)	*Q*
1.8	2242	156 (2164–2320)	0.975	14
5	1199	93 (1152–1245)	0.971	13
9	741	79 (702–781)	0.980	9

**Table 2:** Comparison between simulations and measurements of longitudinal resonance modes with first-order and second-order.

	First-Order Longitudinal Resonance Mode (Abbr. FO)	Second-Order Longitudinal Resonance Mode (Abbr. HO)	FO/HO
Simulated sound pressure (Pa)	0.061	0.0027	22.6
Measured PA signal (mV)	5.345	0.223	24.0

**Table 3:** Performance parameters of STPAC with different tube lengths.

Tube Length (cm)	PA Signal (mV)	Noise Average (mV)	σ (µV)	*SNR*
1.8	1.726	0.8259	2.0	450
5	5.345	0.0563	0.36	14,691
9	9.352	1.5857	1.4	5547

**Table 4:** Performance parameters of STPAC and TPAC.

PAC	PA Signal (mV)	Noise Average (mV)	σ (µV)	*SNR*
STPAC	5.345	0.0563	0.36	14,691
TPAC	0.528	0.0990	0.47	913

**Table 5:** Calibration parameters of STPAC.

Calibration Concentration (ppm)	PA Signal (mV)	Actual Concentration (ppm)	Retrieved Concentration (ppm)	Related Error (%)
0.15	0.166	0.25	0.26	+4
0.5	0.332	2	1.91	−4.5
1	0.593			
5	2.644			
10	5.345			

**Table 6:** Simulated performance parameters of different sphere sizes.

Sphere Diameter (cm)	Sphere Volume (mL)	Resonance Frequency (Hz)	Simulated Sound Pressure (Pa)	Optical Path (cm)
3	14.1	1261	0.0504	182
5.08	68.6	1238	0.0610	308
7	179.5	1234	0.0570	424

**Table 7:** Performance parameters of STPAC and TPAC for CO_2_.

PAC	PA Signal (µV)	Noise Average (µV)	σ (µV)	*SNR*
STPAC	2.362	0.242	0.045	47
TPAC	0.766	0.249	0.051	10

## Data Availability

Data sharing not applicable.
